# Memory Traces Formed in Utero—Newborns’ Autonomic and Neuronal Responses to Prenatal Stimuli and the Maternal Voice

**DOI:** 10.3390/brainsci10110837

**Published:** 2020-11-11

**Authors:** Adelheid Lang, Peter Ott, Renata del Giudice, Manuel Schabus

**Affiliations:** 1 Centre for Cognitive Neuroscience (CCNS), Department of Psychology, University of Salzburg, 5020 Salzburg, Austria; manuel.schabus@sbg.ac.at; 2 Laboratory for Sleep, Cognition and Consciousness Research, University of Salzburg, 5020 Salzburg, Austria; peter.ott@stud.sbg.ac.at (P.O.); renata.delgiudice@asst-santipaolocarlo.it (R.d.G.); 3 Information Technology and Systems Management, Salzburg University of Applied Sciences, 5020 Salzburg, Austria; 4 Department of Mental Health, University of Milan, 20142 Milan, Italy; 5 San Paolo University Hospital, ASST Santi Paolo e Carlo, 20142 Milan, Italy

**Keywords:** perinatal memory, fetal learning, fetus, newborn, maternal voice, heart rate, EEG, speech–brain coupling

## Abstract

In our pilot study, we exposed third-trimester fetuses, from week 34 of gestation onwards, twice daily to a maternal spoken nursery rhyme. Two and five weeks after birth, 34 newborns, who were either familiarized with rhyme stimulation in utero or stimulation naïve, were (re-)exposed to the familiar, as well as to a novel and unfamiliar, rhyme, both spoken with the maternal and an unfamiliar female voice. For the stimulation-naïve group, both rhymes were unfamiliar. During stimulus presentation, heart rate activity and high-density electroencephalography were collected and newborns’ responses during familiar and unfamiliar stimulation were analyzed. All newborns demonstrated stronger speech–brain coupling at 1 Hz during the presentation of the maternal voice vs. the unfamiliar female voice. Rhyme familiarity originating from prenatal exposure had no effect on speech–brain coupling in experimentally stimulated newborns. Furthermore, only stimulation-naïve newborns demonstrated an increase in heart rate during the presentation of the unfamiliar female voice. The results indicate prenatal familiarization to auditory speech and point to the specific significance of the maternal voice already in two- to five-week-old newborns.

## 1. Introduction

The neonatal period is a fascinating stage with rapid developmental changes and major challenges for the newborn. Besides the specific development of the brain structure in this early period [1], newborns need to develop adaptive and self-regulative processes to cope with the highly diverse environment and various kinds of sensory stimulation. In preparation for environmental adaptation and sensory regulation after birth, the nervous and sensory systems in fetuses show enhanced development in the last trimester (for review, see [2]). From a developmental perspective, these processes are furthermore thought to be influenced by individual prenatal experiences and intrauterine conditions [3], also referred to as “prenatal programming”. Moreover, and again for the reason of preparing the unborn baby for its individual extrauterine world, it is suggested that fetuses form memories for recurrent external events to prepare them for future environmental challenges. The process of forming memories in utero is also referred to as fetal learning and was already studied in various ways. It has, for example, been reported that fetuses show distinct heart rate (HR) changes to repeatedly vs. newly presented music [4,5], nursery rhymes, stories, and voices (for an overview, see [6]).

Furthermore, and important to the present study, prenatally formed memory traces are reported to persist into the neonatal period (for review, see [7]). This was, for example, shown in quicker habituation (indicated by decreasing reflex responses) to vibroacoustic stimuli in prenatally vibroacoustic familiarized newborns [8]. However, most evidence comes from the stimulus, which accompanies the fetus’ intrauterine environment from the very beginning onwards, namely the maternal voice itself. Compared to unfamiliar voices, the maternal voice is reported to elicit changes in newborns’ pacifier sucking behavior [9,10] or to decrease movements [11], or is evident as distinct language-specific EEG brain response at birth [12]. What remains to be solved is whether the maternal voice is arousing or calming on an autonomic level, as there are reports about increases [13] as well as decreases [14,15] in infants’ HR, and evidence for reduced sympathetic as well as increased parasympathetic activity [16], during the presentation of the maternal voice.

Newborns’ autonomic responses were also studied for prenatally learned and familiarized vs. unfamiliar stimuli. Granier-Deferre et al. [17], for example, have shown that infants’ HR decreases to prenatally presented music, confirming earlier reports by Hepper [5]. Furthermore, prenatally familiarized newborns show distinct pacifier sucking behavior to familiar vs. novel speech passages [18], likewise reported for the maternal vs. a female stranger’s voice [9,10]. Distinct pacifier sucking patterns were also observed during the presentation of the native vs. a non-native language [19,20], and even identified at a brain level as indicated by increased near-infrared spectroscopy (NIRS) activation to native vs. non-native language [21]. Interestingly, exposure to the native language in utero seems to even shape the “cry melody” in newborns [22], pointing to prenatal language-specific learning. But it was only after about six months of age that native language processing has been linked to greater EEG gamma activity [23]. Other studies in newborns using event-related potentials (ERPs) found larger P350 amplitudes (between 100 and 600 ms) for prenatally familiarized vs. unfamiliar sounds [24] and mismatch responses to prenatally presented pseudowords with unfamiliar pitch changes [25]. Moreover, the authors found generalization to other types of similar, but not prenatally trained, speech sounds. Thus, present evidence suggests that even near-term fetuses might learn speech characteristics including some of the complex spectral and temporal regularities inherent to speech and therefore are likely to learn prosody or the melodic contour of their native language.

To summarize, most of the studies addressing the formation of memories in utero, and performed in newborns, focused on learning paradigms and collected behavioral reactions or changes in autonomic regulation. Consequently, we tried to extend that knowledge and tested (prenatal) memory formation not only on a behavioral level (e.g., by addressing the influence of familiar vs. unfamiliar stimuli on changes in sleep vs. wake behavior of newborns [26]), but additionally focused on newborns’ autonomic regulation (HR) and brain responses (collected with high-density electroencephalography (*hd*EEG)). More specifically, we examined newborns’ physiological responses to nursery rhymes in a group of prenatally familiarized (EG) and stimulation-naïve (CG) newborns. For that purpose, all newborns were exposed to two different nursery rhymes (and only the EG learned one of them before birth), spoken with the maternal vs. an unfamiliar female voice. Given the mixed results in the literature, we analyzed the HR responses in two time windows and expected lower initial HR (stronger initial orienting response [27]) as well as lower HR over stimulation time when familiar material (familiar voice as well as familiar rhyme) was presented, as we expected that familiarity is rather calming than arousing by nature. Furthermore, we expected stronger speech–brain coupling to the familiar maternal voice as well as to the familiar rhyme (in the EG).

## 2. Materials and Methods

### 2.1. Participants

Fifty-five mothers-to-be were recruited at information evenings for parents-to-be of local hospitals in Salzburg/Austria. Due to premature birth or various pregnancy-related problems, 10 participants of the initial sample dropped out. From the 45 infants finishing the full study, we later excluded ECG data from 11 infants (EG = 9; CG = 2) and EEG data from 8 infants (EG = 7, CG = 1) due to noncorrectable artifacts (muscle artifacts and/or bad electrodes). The final sample consisted of 34 infants (EG = 22; CG = 12) for heart rate analyses, and 37 infants (EG = 24; CG = 13) for speech–brain coupling. Mean age in infants was 14.35 days (*SD* = 2.67) in the first recording and 36.48 days (*SD* = 3.43) in the second recording. All infants in our final sample were born healthy and full-term (>38 weeks of gestation, *M* = 39.31). At birth, the mean age of the mothers was 31.91 years (*SD* = 4.84) and all of them were native German-speaking; 52% were married, 48% were living with their partner, and 36% held a university degree. The presented study was approved by the ethics committee of the University of Salzburg (EK-GZ 12/2013) and participants gave written informed consent at the first appointment (<34 weeks of gestation).

### 2.2. Materials

For auditory stimulation we used two German nursery rhymes, which were distinct in rhythm (lively vs. calm). The distinct rhythms were chosen for the reason of presenting well-distinguishable rhymes even for newborns, but the rhythm of the rhymes were not from interest for our hypotheses. For every single subject, we taped both rhymes with the maternal, and therefore “familiar”, voice, but also with an unfamiliar female voice (a professional female speaker). To standardize the total length of each rhyme to exactly 60 s, we used the software Audacity^®^ (iWeb Media Ltd., Birkirkara, Malta [28]). Twice daily (morning and evening), mothers-to-be were asked to run the prenatal stimulation protocol (replaying the specified rhyme) for five minutes (i.e., five repetitions of the originally recorded 60 s rhyme). For stimulation after birth, familiar and unfamiliar stimuli were presented in random order over loudspeakers (60 db) and using Presentation^®^ (NeuroBehavioral Systems, Berkeley, CA, USA [29]).

### 2.3. Experimental Procedure

**Figure 1:**
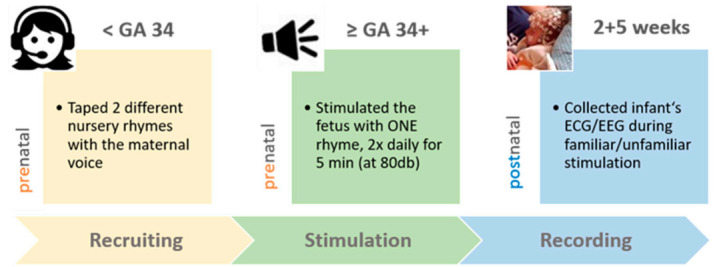
Experimental procedure. Mothers-to-be (*n* = 34; fetus < GA34) taped two different nursery rhymes during a visit in our laboratory and were afterwards assigned (randomly) to two groups. In the experimental group (EG, *n* = 22), one nursery rhyme was replayed to the fetus (80 dB over loudspeakers; presented twice daily for five minutes) the last six weeks (>GA34) until birth. In the control group (CG, *n* = 12), fetuses were not stimulated with any rhyme. After birth (two and again five weeks) EG and CG infants’ electrocardiography (ECG) and high-density electroencephalography (*hd*EEG) were collected during auditory stimulation with both rhymes presented with both voices, namely the familiar (maternal) and an unfamiliar female voice. GA = gestational age.

Our experimental protocol included a prenatal and postnatal stimulation part (see Figure 1). For the prenatal stimulation part, expecting mothers (<34 weeks of gestation) taped the two different nursery rhymes during a visit in our laboratory. The experimental group (EG; *n* = 22) received a CD with either the lively or the calm rhyme (randomly chosen), taped with the maternal voice, and replayed it to the third-term fetus over speakers (with 80 dB as dampening of about 20 db is expected across the mother’s belly [30]), from gestational week 34 until the day of birth. Fetuses were stimulated in the maternal home environment twice daily (in the morning and the evening) for five minutes. During prenatal auditory stimulation, mothers-to-be were asked to relax and sit down in a quiet room, but also to avoid touching their belly. With a provided tablet, they also documented the number of daily stimulations and the sound pressure level during the stimulations, as well as their subjective well-being. An additional control group (*n* = 12) visited our laboratory to tape the same two nursery rhymes but did not replay any rhyme to their unborn baby. After birth, these mothers and newborns completed the same protocol.

After birth (two as well as five weeks later), our team visited the majority of the participating families in their home environment (only four families preferred to visit our lab for postnatal recordings). After setting up the equipment (EEG system, speakers, camera, and a laptop for presenting the stimuli), instructing the mothers, and explaining the next steps, an *hd*EEG cap (Electrical Geodesic Inc., Eugene, OR, USA [30]) and electrodes for ECG measures were carefully placed on the infant. Afterwards, the infant’s video, ECG, and *hd*EEG were continuously collected during auditory stimulation and intermediate baseline (silence) periods. Auditory stimulation included the two taped nursery rhymes, both spoken with the maternal (familiar) voice and with an unfamiliar (professional female speaker) voice. Each of the two postnatal recordings lasted 27 min. The four different stimuli (2 rhymes × 2 voices) were presented in a randomized order and with a duration time of three minutes (=three repetitions of the 60 s lasting rhyme) for each stimulus. Before the presentation of the first stimulus, as well as between all stimuli and after the last stimulus, we added a three-minute baseline (silence). The postnatal experimental setting was identical at two and five weeks after birth and identical for both groups (experimental and control group).

### 2.4. Electrophysiological Data Collection

For EEG acquisition, we used a 128-electrode GSN HydroCel Geodesic Sensor Net (Electrical Geodesic Inc., Eugene, OR, USA [31]) that matched with the infant’s head circumference (available net sizes: 34–36, 36–37, 37–38 cm), and a Net Amps 400 amplifier (Electrical Geodesic Inc., Eugene, OR, USA [31]). For ECG measurements, we placed two additional electrodes above the infant’s right clavicle and its left abdomen. EEG and ECG data were both recorded with a sampling rate of 1000 Hz. To keep the infants as calm as possible while collecting electrophysiological data, they were either lying still in their mother’s arms or were only gently rocked if needed. 

### 2.5. ECG Preprocessing and Analysis

ECG data were preprocessed with Matlab (The MathWorks Inc., Natick, MA, USA [32]) software using the Anslab Professional [33] toolbox. After manually correcting for incorrectly detected R-peaks and artifacts, mean heart rates were calculated for the following epochs of interest. To analyze the orienting response (OR) for the four different stimulus types (two rhymes, each presented with two different voices), mean HR was calculated in beats per minute for the first 10 s after stimulus onset (according to Richards and Casey [27]), as well as the last 10 s of the respective preceding baselines. To analyze the stimulation response (SR), mean HR was calculated for the full 180 s presentation time per stimulus type and the respective 180 s preceding baselines. Afterwards, the change from baseline to stimulation was calculated for both time windows, by subtracting mean HR during preceding baselines from mean HR during the four different corresponding stimulation phases.

### 2.6. Speech–Brain Coupling

*hd*EEG data was recorded with 1000 Hz, downsampled to 125 Hz, high-pass filtered at 0.5 Hz (Butterworth with stopband attenuation −80 db at 0.05 Hz and 10^−6^ db ripple at 0.5 Hz), and notch filtered at 50 Hz (±1 Hz; 10th order Butterworth). Artefacts were removed using standard methods (exclusion of bad channels, channel interpolation, grand-average re-referencing, and independent component analysis to remove remaining artefacts). To investigate speech–brain coupling, we used the Fieldtrip toolbox [34] in Matlab (The MathWorks Inc., Natick, MA, USA [31]) and adopted the method from Gross and colleagues [35] for our 128-channel *hd*EEG HydroCel Geodesic sensor nets. At first, we computed the speech amplitude envelope of the prerecorded voice data (adopted from Gross et al. [35]; nine cochlear bands (100–1000 Hz) with 10th order Butterworth; absolute Hilbert envelope for each cochlear band; average of Hilbert envelopes). As a next step, we filtered the amplitude envelope data and the EEG data to arrive at the desired target bands at syllable, word, and sentence level (4 Hz, 2 Hz, and 1 Hz; ±0.5 Hz; 4th order Butterworth). Mutual information (MI) was then used as a coupling metric for each *hd*EEG channel and pooled for a temporal region of interest (cf. Supplementary Materials Figure S1), together with the speech envelope. The entropy-based MI uses the signal complexity as a measure and better accounts for (remaining) artefacts as compared to simpler methods like frequency coherence. Coherence analysis of amplitude coupling among speech envelope EEG channels was done on three-second segments focusing on the delta, theta, alpha, and beta bands. For deriving reliable estimates of the coupling matrix, we decided to combine the recordings of week two and week five of each newborn (i.e., always two recordings of the same subjects).

### 2.7. Statistical Analyses

For statistical analyses we used IBM SPSS Statistics 26 (IBM, Armonk, NY, USA [36]) and repeated measures and mixed analyses of variance (ANOVA). Significance levels were set to *p* < 0.05. Effect sizes are reported as partial eta squared (*p.eta*^2^), with 0.01 considered as small, 0.06 as medium, and 0.14 as large effect size, and as Cohen’s d (*d*), with 0.2–0.3 interpreted as small, ≈0.5 as medium, and >0.8 as large effect size. In case of violations of sphericity, Greenhouse–Geisser correction was utilized. To test how the newborns’ HR changed depending on voice familiarity (maternal vs. unfamiliar female voice), we computed two ANOVAs focusing on the VOICE familiarity effect with the factors AGE × VOICE × GROUP (EG, CG) and calculated the HR change (in beats per minute) from the preceding baselines to the stimulation periods with the maternal and the unfamiliar voice, and for both time windows of interest (10 sec OR, 180 sec SR). 

To examine the familiarity effect of the prenatally presented rhyme in the EG, we repeated the above described procedure for OR and SR but split the stimulation periods into familiar and unfamiliar rhyme. We therefore computed two ANOVAs for the EG with the factors AGE × VOICE × RHYME for the OR as well as the full stimulation period (SR). Please note: In order to keep the result section concise and focused, we are only reporting the significant main effects and interactions of interest, as well as statistical trends (*p* < 0.10), and omitting nonsignificant effects.

Additionally, we performed an exploratory analysis and checked whether the nature of the rhyme (calm vs. lively) has an effect on infants’ (EG and CG) HR changes (see Supplementary Materials Figure S4). For speech envelope to EEG amplitude coupling, mutual information was used as the coupling metric on the Hilbert-transformed amplitude data, and analyzed in the three frequency bands at a “syllable” (4 Hz), “word” (2 Hz), and “sentence” level (1 Hz) according to Gross et al. [35]. Consequently, we here corrected for multiple comparisons and only report results surviving a *p*-level of <0.0166.

## 3. Results

### 3.1. Effect of Rhyme Familiarity on Infant’s Heart Rate (EG)

Repeated measures ANOVA with the within-factors AGE (2, 5 weeks), RHYME (familiar vs. unfamiliar), and VOICE (mother vs. female stranger) revealed no significant main effects and interactions. The prenatal replayed rhyme neither influenced the HR in the early orienting response time window (OR; *F*(1, 21) = 0.01, *p* = 0.972, *p.eta*^2^ = 0.00; cf. Supplementary Materials Figure S2) nor the HR during the subsequent time window of stimulus presentation (SR; *F*(1, 21) = 1.22, *p* = 0.281, *p.eta*^2^ = 0.06; cf. Supplementary Materials Figure S3) in the (in utero stimulated) EG. For exploratory analyses of how the nature of rhyme (calm vs. lively) influenced OR and SR in the EG, but also in the CG, please refer to Supplementary Materials Figure S5 and S6

### 3.2. Effect of Voice Familiarity on Infant’s Heart Rate (EG and CG)

Repeated measures ANOVA with the within-factors AGE (2, 5 weeks) and VOICE (mother vs. female stranger), and the between-factor GROUP (EG vs. CG) revealed no significant main effects or interactions. The maternal voice elicited no specific OR in infants with (EG) or without (CG) prenatal stimulation within the first 10 s of stimulation, as indicated by a nonsignificant main effect for VOICE (*F*(1, 32) = 0.10, *p* = 0.750, *p.eta*^2^ = 0.01; for the EG cf. Supplementary Materials Figure S2) and a nonsignificant interaction for VOICE*GROUP (*F*(1, 32) = 1.40, *p* = 0.246, *p.eta*^2^ = 0.042).

**Figure 2:**
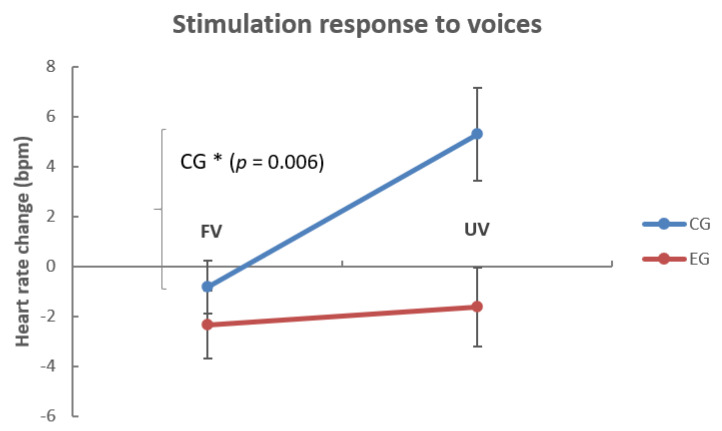
Change in HR (beats per minute) in dependence of voice familiarity. The figure shows the HR change (in beats per minute) from (4×) 180 s of the preceding baseline to (4×) 180 s stimulus presentation in infants who were prenatally exposed to auditory stimulation (EG; *n* = 22) and who were not exposed (CG; *n* = 12). Note that only in the CG did the HR increase to the unfamiliar voice, with respect to the HR change in the EG, that is not significantly different from baseline. HR = heart rate, EG = experimental group, CG = control group, FV = familiar voice, UV = unfamiliar voice. Data were pooled for both recordings two and five weeks after birth. Error bars refer to +/− 1 SEM.

To analyze the SR (0–180 s) we calculated a repeated measures ANOVA for the corresponding time window of interest with the within-factors AGE (2, 5 weeks) and VOICE (mother vs. female stranger). As the naïve CG was not stimulated before birth, we again additionally included the between-factor GROUP (EG, CG) in our analyses. We found significant main effects for VOICE (*F*(1, 32) = 4.23, *p* = 0.048, *p.eta*^2^ = 0.12) and GROUP (*F*(1, 32) = 7.09, *p* = 0.012, *p.eta*^2^ = 0.18; cf. Figure 2) but no VOICE x GROUP (*F*(1, 32) = 2.66, *p* = 0.112, *p.eta^2^* = 0.08) or VOICE × GROUP × AGE interaction (*F*(1, 32) = 0.12, *p* = 0.728, *p.eta*^2^ = 0.01). Post hoc paired t-test revealed that only in the CG (*t*(11) = −3.36, *p* = 0.006, *d* = 1.13), but not in the EG (*t*(21) = −0.32, *p* = 0.755, *d* = 0.09), infants’ mean HR was increased (in comparison to the baseline; cf. Figure 2) during the presentation of the unfamiliar voice (*M* = 5.29, *SE* = 1.84), but not in response to the familiar voice (*M* = −0.82, *SE* = 1.06). For the individual HR response to the presented voices, please refer to Supplementary Materials Figure S4. We added an exploratory analysis of whether the type of RHYME (calm vs. lively) played a role in the CG’s HR increase and found the trend that especially the lively rhyme in the unfamiliar voice led to HR increase (cf. supplemental material and Supplementary Materials Figure S5). In summary, only infants in the auditory stimulation-naïve CG were aroused by the unfamiliar female voice (cf. Figure 2), and especially if it was paired with the lively rhyme (cf. Supplementary Materials Figure S5).

### 3.3. Effect of Rhyme Familiarity on Infant’s Brain Physiology (EEG; EG)

To analyze whether the recognition of prenatally learned material is evident in the newborn at brain level, we performed a speech-envelope coupling analysis of *hd*EEG signals recorded at two and five weeks after birth (here, two recordings per baby were pooled for analysis). Repeated measures ANOVA with the within-factors RHYME (familiar vs. unfamiliar) and FREQ (frequency bands; 1, 2, and 4 Hertz) revealed the interaction RHYME × FREQ (*F*(2, 46) = 6.16, *p* = 0.004, *p.eta*^2^ = 0.21). Post hoc paired *t*-tests have shown no significant effects after correcting for multiple comparisons (*p* > 0.0166; cf. Supplementary Materials Table S1 for descriptive measures). 

### 3.4. Effect of Voice Familiarity on Infant’s Brain Physiology (EEG; EG and CG)

**Figure 3:**
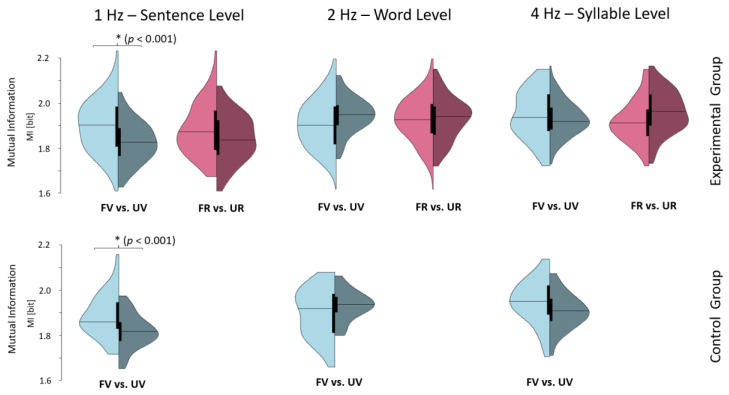
Speech-envelope amplitude coupling with EEG. The horizontal line indicates the mean mutual information (in Bit units) for a spatial cluster around the temporal cortices (cf. Supplementary Materials Figure S1). Note that the infants’ brains couple more to the familiar mother’s voice (FV) as compared to an unfamiliar voice (UV) at 1 Hz. This distinct mutual information difference is seen for both groups, indicating a strong preference for the maternal voice at birth independent of whether fetuses were daily stimulated with the mother’s voice. Note: For the control group, only the voice effect is illustrated as there is no familiarization to a rhyme (FR) before birth. FR = familiar rhyme; UR = unfamiliar rhyme; FV = familiar voice; UV = unfamiliar voice. * *p* < 0.001.

Repeated measures ANOVA with the within-factors VOICE (mother vs. female stranger) and FREQ (frequency bands; 1, 2, and 4 Hertz) and the between-factor GROUP (EG vs. CG) revealed a main effect for VOICE (*F*(1, 36) = 9.39, *p* = 0.004, *p.eta*^2^ = 0.21) and a significant interaction effect for VOICE x FREQ (*F*(2, 72) = 39.15, *p* < 0.001, *p.eta*^2^ = 0.52) but no interactions with GROUP (p’s > 0.402). Post hoc paired t-test indicates higher coupling of newborns’ oscillatory brain activity to the mothers as compared to the unfamiliar female voice in both groups (EG and CG; cf. Figure 3) at 1 Hz (CG: *t*(12) = 6.06, *p* < 0.001, *d* = 1.64; EG: *t*(23) = 5.70, *p* < 0.001, *d* = 1.22). Altogether, the brain-level data indicate a distinct response of the newborn’s brain to whether stimulus (re-)exposure contains the familiar mother or unfamiliar female voice, independent of prenatal auditory stimulation.

## 4. Discussion

The present study aimed to investigate whether newborns show signs of recognition to prenatally presented nursery rhymes, observable on an autonomic (HR) and neuronal (speech–brain coupling) level. We hypothesized that, in comparison to a newly presented nursery rhyme, the prenatally familiarized rhyme elicits a stronger orienting response (transient decrease in HR) and a distinct HR reaction over stimulation time, as well as stronger speech–brain coupling. Furthermore, we hypothesized a similar pattern for the familiar maternal voice as compared to another unfamiliar female voice in all newborns, irrespective of prenatal stimulation. 

Contrary to earlier reported distinct behavioral reactions to prenatally presented stimuli [5,17,18], we found no clear evidence for stimulus recognition of the prenatally replayed nursery rhyme. We also did not find stronger speech–brain coupling for the familiar vs. the unfamiliar rhyme, which is not in line with earlier studies, reporting stronger brain activation to prenatally familiarized speech stimuli [25] and sounds [24]. Given our current analysis, we conclude that prenatally formed memories for complex material such as nursery rhymes are only encoded to a very basic degree, which is not identifiable at a behavioral [26] or physiological level (ECG and EEG). We still believe that bigger samples and more controlled studies (e.g., for behavioral states during stimulus presentation) could reveal a small effect even for such complex speech material and believe that it is worth intensifying work in that direction. In addition, it is important to note that in our study, infants were tested very early in age (two and five weeks after birth) and at this age range, stimulation is taking place most of the time during sleep periods which will diminish the observable effects. Still, especially for speech–brain coupling, it is reported that even in “offline” sleep periods, the brain couples distinctly to meaningful vs. irrelevant speech stimuli [37], at least in the adult brain.

Besides the negative finding for rhyme familiarity, brain-level data clearly indicate that the maternal voice is “easier” to track by the infant’s brain as indicated by stronger speech–brain coupling (mutual information) for the familiar mother’s voice. This is also in line with a former study, reporting a distinct brain activation for the maternal vs. an unfamiliar voice [12]. 

Interestingly, and in contrast to former studies focusing on behavioral reactions [10,11,31], newborns did not show a stronger orienting response and HR decrease to the maternal voice. However, infants in the auditory stimulation-naïve CG were more aroused over the stimulation time with the unfamiliar female voice, especially if the voice was paired with the lively (and therefore likely arousing) rhyme, as was evident in increased mean heart rates. We found a related result in an earlier study analysis, focusing on the effects of auditory stimulation on changes in sleep–wake states [26]. In that study, the prenatal familiarized EG was calming down over stimulation time, irrespective of rhyme or voice, and showed a higher proportion of (especially quiet or “deep”) sleep. In the auditory stimulation-naïve CG, this generalized calming effect of rhyme replay just after birth was completely lacking. Together with these earlier findings, we conclude the CG is more “alerted” in the novel auditory-stimulation situation where (unfamiliar) rhymes are replayed to them over speakers. We therefore conclude that the observed increase in HR in response to the unfamiliar voice is likely the result of, in general, more attention to new stimuli and especially more arousal to unfamiliar stimuli, such as a nursery rhyme spoken by the voice of a stranger.

We are aware that our preliminary findings are limited by the sample size and the unbalanced group sizes in the EG and CG. Yet using physiological measures (*hd*EEG together with ECG) in the habitual home environment of healthy newborns is a huge challenge in itself. Firstly, it takes a lot of effort to visit parents at home, set up the ambulant EEG system, and prepare the newborn with ECG electrodes and EEG nets in such short time periods that the young participants are not already exhausted before the study protocol can start. Secondly, infants at two but also at five weeks of age have no stable sleep–wake rhythm and are strongly fluctuating in wake behavior as well as basic needs such as eating. 

In conclusion, our preliminary results indicate that newborns show distinct reactions to the maternal voice already at birth (two and five weeks) even on a physiological level and identifiable with ECG and EEG. Furthermore, it appears that basic memory traces are formed in utero and shape the newborn’s autonomic and neuronal reactions to speech and voice stimuli, namely, in such a way that newborns familiarized to nursery rhymes prenatally show distinctly different reactions than newborns being naïve in this respect. This again emphasizes the importance of the prenatal environment and calls into attention that already at these times the brain is tuned or “programmed” for the postnatal environment predicted and most likely experienced.

## Supplementary Materials

The following are available online at https://www.mdpi.com/2076-3425/10/11/837/s1, Figure S1: Temporal clusters for speech-envelope coupling analysis; Figure S2: Orienting response (0–10 s) to rhyme and voice familiarity; Figure S3: Stimulation response (0–180 s) to rhyme and voice familiarity; Figure S4: Individual stimulation response (0–180 s) to voices; Figure S5: Orienting response to nature of rhyme and voice; Figure S6: Overall change in HR to nature of rhyme and voice; Table S1: Means and standard deviations for voice/rhyme stimuli in different frequency bands.
