# Supplementary material for: Memory Traces Formed in Utero—Newborns’ Autonomic and Neuronal Responses to Prenatal Stimuli and the Maternal Voice
Source: Brain Sci. 2020 Nov 11;10(11):837. doi: 10.3390/brainsci10110837 (PMC7697227; doi:10.3390/brainsci10110837)
Supplement: Supplementary file 1 [file brainsci-10-00837-s001.pdf]

Memory traces formed in utero – Newborn's autonomic and neuronal responses to prenatal stimuli and the maternal voice

Adelheid Lang [1,2,*], Peter Ott, [2,3], Renata del Giudice [2,4,5] and Manuel Schabus [1,2,*]

[1] Department of Psychology, Centre for Cognitive Neuroscience (CCNS), University of Salzburg, 5020 Salzburg, Austria

[2] Laboratory for Sleep, Cognition and Consciousness Research, University of Salzburg, 5020 Salzburg, Austria

[3] Information technology and systems management, Salzburg University of Applied Sciences, 5020 Salzburg, Austria

[4] Department of Mental Health, University of Milan, 20142 Milan, Italy

[5] San Paolo University Hospital, ASST Santi Paolo e Carlo, 20142 Milan, Italy

* Correspondence: adelheid.lang@sbg.ac.at and manuel.schabus@sbg.ac.at; Tel.: +43-662-8044-5137

Supplemental Files

Methods

Here we illustrate the temporal clusters used for the speech-envelope-coupling analysis.

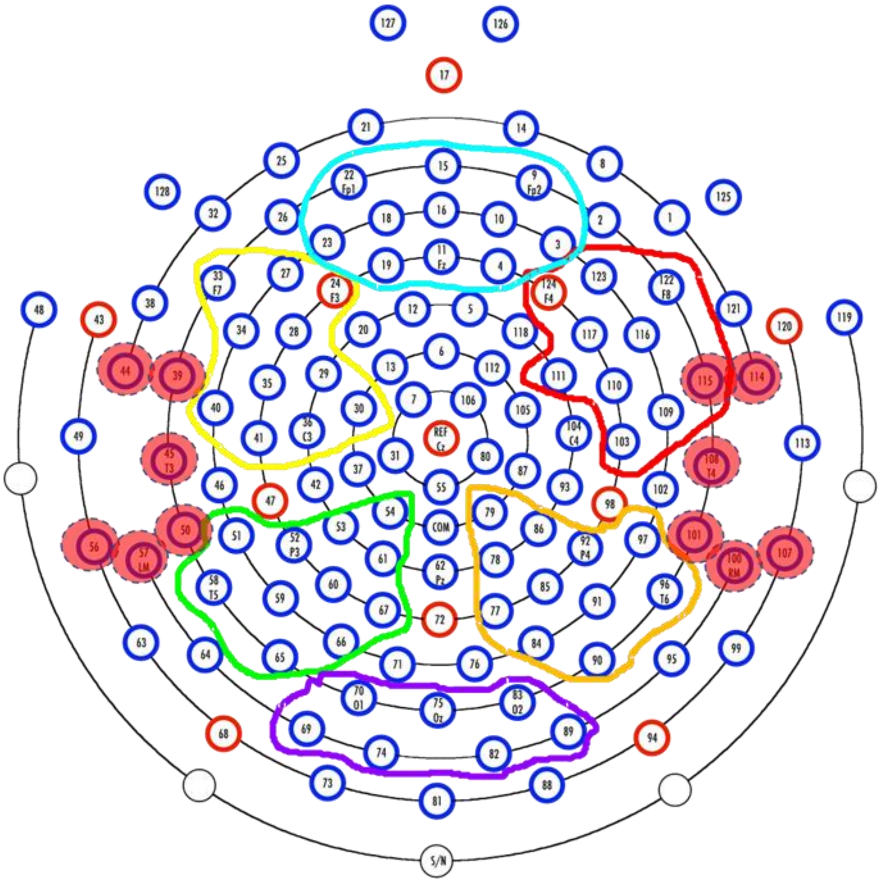

Figure S1. **Temporal clusters for speech-envelope-coupling analysis.** The red dots mark the temporal electrodes we used for analysing speech-envelope-coupling. These electrodes are the electrodes best overlaying the temporal cortices in the recorded sample of two and five week old babies.

Results

Please find exploratory, or more detailed, analyses in the following section.

*Effect of **rhyme** and **voice familiarity** on infant's **heart rate** (EG)*

Infant's HR did not change specifically to the prenatally presented (familiar) rhyme or the newly presented (unfamiliar) rhyme or to the combination of rhyme and voice familiarity. Neither in the 10 seconds orienting response nor in the 180 seconds stimulation response.

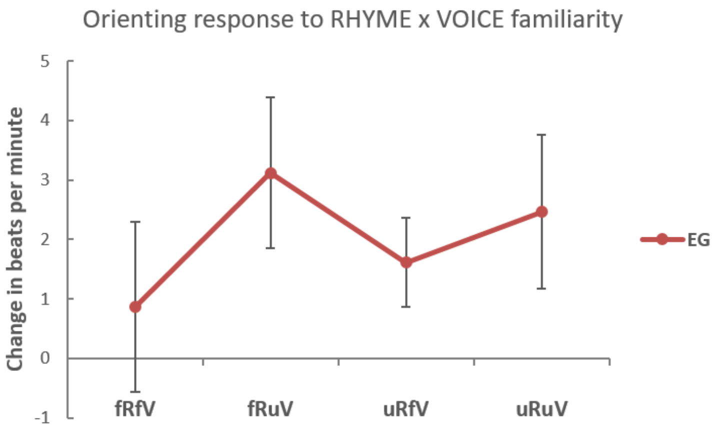

Figure S2. **Orienting response** (0-10 sec**) to rhyme and voice familiarity.** Neither the prenatally repeatedly presented rhyme (**fR**), nor the maternal voice (**fV**) elicited a specific 10 seconds orienting response in infant's HR change from baseline to stimulation. Data are pooled from the same babies recorded twice at week 2 and week 5 of age. fR = familiar rhyme, uR = unfamiliar rhyme, fV = familiar voice, uV = unfamiliar voice, EG = experimental

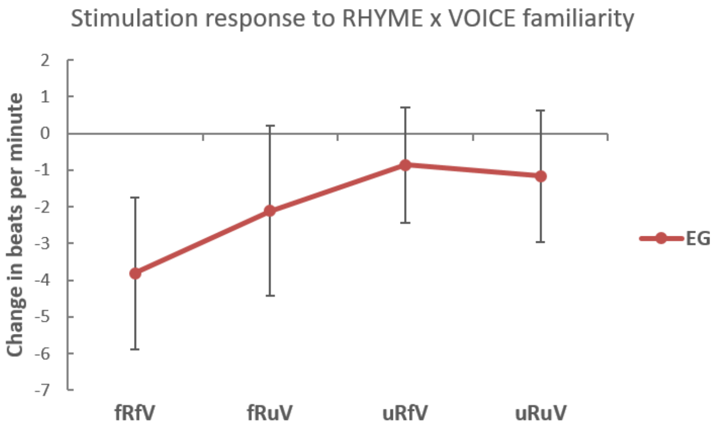

Figure S3. **Stimulation response** (0-180 sec) **to rhyme and voice familiarity.** Neither the prenatally repeatedly presented rhyme (**fR**), nor the maternal voice (**fV**) elicited a specific 180 seconds stimulation response in infant's HR change from baseline to stimulation. Data are pooled from the same babies recorded twice at week 2 and week 5 of age. fR = familiar rhyme, uR = unfamiliar rhyme, fV = familiar voice, uV = unfamiliar voice, EG = experimental group. Error bars = +/- 1 SEM.

Individual heart rate response *to the maternal and unfamiliar voice*

Please find the individual heart rate response to the stimulation with familiar and unfamiliar voices below. As mentioned in the main text of the manuscript, former studies reported mixed findings (in- or decreases in heart rate) during the presentation of the maternal voice. In our study, infant's heart rates to the maternal and to an unfamiliar voice are not systematically changing.

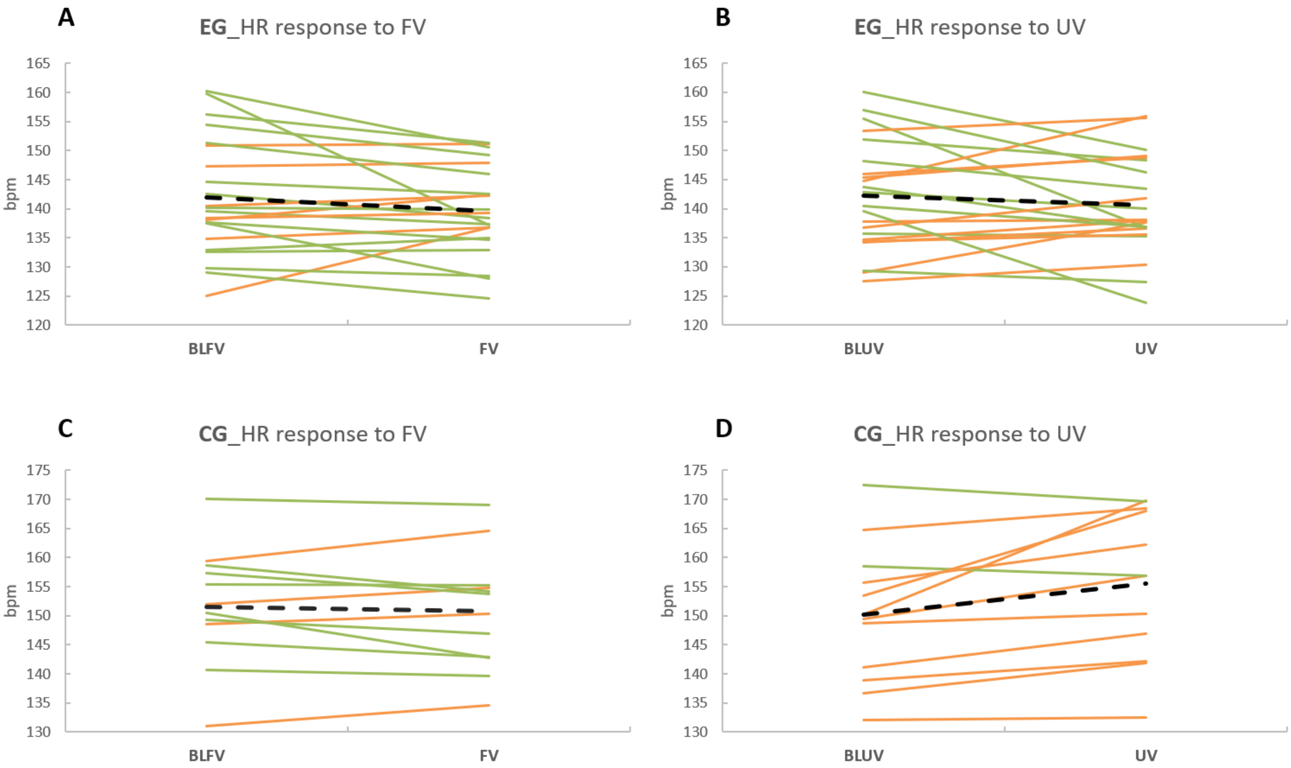

Figure S4. **Individual stimulation response** (0-180 sec) **to voices.** Newborn's have shown a mixed pattern of heart rate increases (in orange) as well as decreases (in green) to the maternal voice (for EG refer to **A**; for CG refer to **C**) and the unfamiliar female voice (for EG refer to **B**; for CG refer to **D**). The black lines indicate the group means. Data are pooled from the same infants recorded twice at week 2 and week 5 of age. BL = baseline (silence), fV = familiar voice, uV = unfamiliar voice, EG = experimental group, CG = control group, bpm= HR in beats per minute, HR = heart rate.

Effect of **RHYME** (calm vs. lively) on infant's **heart rate** (EG and CG)

Mixed ANOVA with the within-factors AGE (2, 5 weeks), VOICE (mother vs. female stranger), the exploratory within-factor nature of RHYME (calm vs. lively) and the between-factor GROUP (EG vs. CG) revealed no significant main effects or interactions for the orienting response (OR; cf. *Figure S5*).

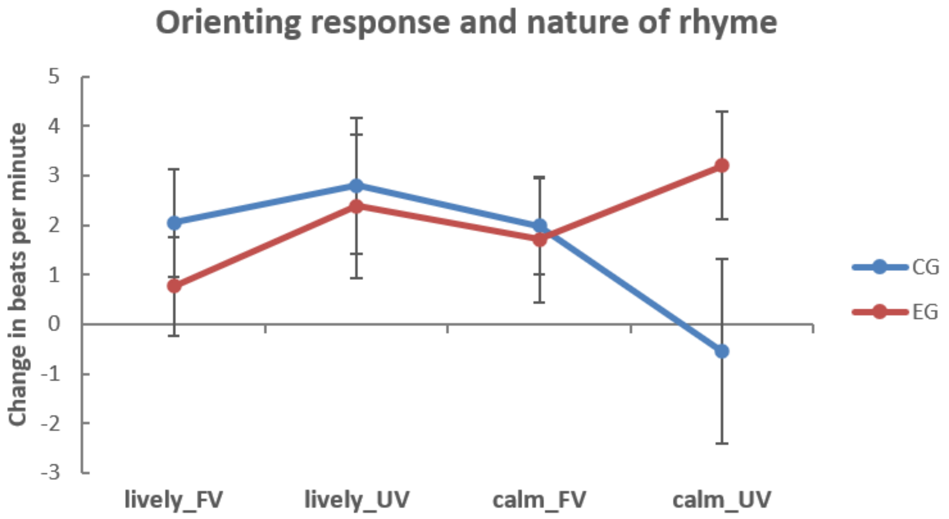

Figure S5. **Orienting response to nature of rhyme and voice.** The figure shows the HR change from 10 seconds of the preceding baseline to (4x) 10 seconds stimulus presentation in infants who were prenatally exposed to auditory stimulation (EG; n=22) and who were not exposed (CG; n=12). Note that groups show no difference in HR change to the voices (FV, UV) or the rhymes (calm vs. lively). Only the calm rhyme, presented with the unfamiliar voice, induced a distinct response in the EG and CG. EG = experimental group, CG = control group, calm = calm rhyme, lively = lively rhyme, FV = familiar voice, UV = unfamiliar voice. Data are pooled for both recordings two and five weeks after birth. Error bars refer to +/- 1 SEM.

On the contrary, infant's heart rate over stimulation time (SR) was influenced by the nature of RHYME ($F(1, 32) = 4.52$, $p = 0.041$, $p.eta^2 = 0.12$). Together with the significant main effects for VOICE ($F(1, 32) = 4.23$, $p = 0.048$, $p.eta^2 = 0.12$) and GROUP ($F(1, 32) = 7.09$, $p = 0.012$, $p.eta^2 = 0.18$) we were interested, how these factors influenced mean HR in the EG and CG (cf. *Suppl. Figure 6*). Post-hoc independent t-test revealed that the lively ($t(32) = 2.33$, $p = 0.026$, $d = 0.84$) as well as the calm rhyme ($t(32) = 1.77$, $p = 0.087$, $d = 0.64$), both presented with the unfamiliar voice, induced a different response in the EG and the CG. Only in the CG the HR increased to the lively ($M = 7.42$, $SE = 2.32$), as well as the calm rhyme ($M = 3.15$, $SE = 3.21$) presented with the unfamiliar voice.

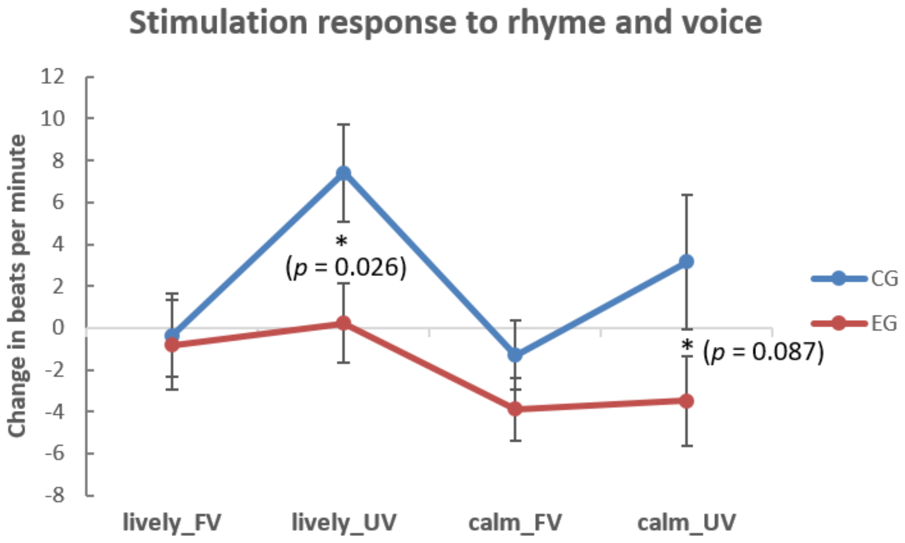

Figure S6. **Overall change in HR to nature of rhyme and voice.** The figure shows the HR change from 180 seconds of the preceding baseline to (4x) 180 seconds stimulus presentation in infants who were prenatally exposed to auditory stimulation (EG; n=22) and who were not exposed (CG; n=12). Note that groups show no difference in HR change to the maternal voice (FV), but to the unfamiliar voice (UV). Only HR in the CG increases to the UV, regardless of the nature of rhyme. EG = experimental group, CG = control group, calm = calm rhyme, lively = lively rhyme, FV = familiar voice, UV = unfamiliar voice. Data are pooled for both recordings two and five weeks after birth. Error bars refer to +/- 1 SEM.

Descriptive measures for **speech-brain coupling**

Please find below a table with the mean MI (mutual information) and SD for both groups (EG and CG) and voice / rhyme familiarity in all frequency bands of interest.

Table S1. *Means and standard deviations for voice/rhyme stimuli in different frequency bands*

Note. M and *SD* are used to represent means and standard deviations of mutual information to familiar (fam) and unfamiliar (unfam) voices and rhymes.

			EG		CG	
**Stimulus**	**Familiarity**	**Frequency**	***M***	***SD***	***M***	***SD***
Voice	fam	1 Hz	1.896	0.073	1.882	0.047
	unfam		1.821	0.036	1.816	0.032
	fam	2 Hz	1.914	0.071	1.892	0.058
	unfam		1.949	0.040	1.935	0.042
	fam	4 Hz	1.953	0.052	1.941	0.056
	unfam		1.939	0.028	1.904	0.031
Rhyme	fam	1 Hz	1.875	0.059	N/A	N/A
	unfam		1.843	0.060	N/A	N/A
	fam	2 Hz	1.930	0.063	N/A	N/A
	unfam		1.935	0.047	N/A	N/A
	fam	4 Hz	1.928	0.054	N/A	N/A
	unfam		1.964	0.040	N/A	N/A
